# TreeBUGS: An R package for hierarchical multinomial-processing-tree modeling

**DOI:** 10.3758/s13428-017-0869-7

**Published:** 2017-04-03

**Authors:** Daniel W. Heck, Nina R. Arnold, Denis Arnold

**Affiliations:** 1 Department of Psychology, School of Social Sciences, University of Mannheim, Schloss EO 266, D-68131 Mannheim, Germany; 2 Quantitative Linguistics, Eberhard Karls University, Tübingen, Germany; 3 Institut für Deutsche Sprache, Mannheim, Germany

**Keywords:** Multinomial modeling, Individual differences, Hierarchical modeling, R package, Bayesian inference

## Abstract

Multinomial processing tree (MPT) models are a class of measurement models that account for categorical data by assuming a finite number of underlying cognitive processes. Traditionally, data are aggregated across participants and analyzed under the assumption of independently and identically distributed observations. Hierarchical Bayesian extensions of MPT models explicitly account for participant heterogeneity by assuming that the individual parameters follow a continuous hierarchical distribution. We provide an accessible introduction to hierarchical MPT modeling and present the user-friendly and comprehensive R package TreeBUGS, which implements the two most important hierarchical MPT approaches for participant heterogeneity—the beta-MPT approach (Smith & Batchelder, *Journal of Mathematical Psychology 54*:167-183, 2010) and the latent-trait MPT approach (Klauer, *Psychometrika 75*:70-98, 2010). TreeBUGS reads standard MPT model files and obtains Markov-chain Monte Carlo samples that approximate the posterior distribution. The functionality and output are tailored to the specific needs of MPT modelers and provide tests for the homogeneity of items and participants, individual and group parameter estimates, fit statistics, and within- and between-subjects comparisons, as well as goodness-of-fit and summary plots. We also propose and implement novel statistical extensions to include continuous and discrete predictors (as either fixed or random effects) in the latent-trait MPT model.

Multinomial processing tree (MPT) models are a class of measurement models that estimate the probability of underlying latent cognitive processes on the basis of categorical data (Batchelder & Riefer, 1999). MPT models make the underlying assumptions of a psychological theory explicit, are statistically tractable and well understood, and are easily tailored to specific research paradigms (for reviews, see Batchelder & Riefer, 1999; Erdfelder et al., 2009). Moreover, recent developments have allowed for modeling the relative speed of cognitive processes in addition to discrete responses within the MPT framework (Heck & Erdfelder, 2016; Hu, 2001).

Traditionally, MPT models are fitted using data aggregated across participants (i.e., summed response frequencies) to obtain a sufficiently large number of observations for parameter estimation and for a high statistical power of goodness-of-fit tests. However, the aggregation of data is only justified under the assumption that observations are identically and independently distributed (i.i.d.) for all participants and items. In case of heterogeneity of participants or items, these conditions are violated, which might result in biased parameter estimates and incorrect confidence intervals (e.g., Klauer, 2006; Smith & Batchelder, 2008, 2010). Moreover, fitting separate models per participant is often not possible due to insufficient numbers of individual responses, which prevents a reliable estimation of model parameters. In recent years, several approaches have been developed to account for heterogeneity in MPT models. Here, we focus on hierarchical Bayesian MPT models that explicitly assume separate parameters for each participant, which follow some continuous, hierarchical distribution on the group level (Klauer, 2010; Smith & Batchelder, 2010).

MPT models are very popular and widely used in many areas of psychology (Batchelder & Riefer, 1999; Erdfelder et al., 2009; Hütter & Klauer, 2016). Partly, this success may be due to the availability of easy-to-use software packages for parameter estimation and testing goodness-of-fit such as AppleTree (Rothkegel, 1999), GPT (Hu & Phillips, 1999), HMMTree (Stahl & Klauer, 2007), multiTree (Moshagen, 2010), and MPTinR (Singmann & Kellen, 2013). For psychologists who are primarily interested in substantive research questions, these programs greatly facilitate the analysis of either individual or aggregated data. They allow researchers to focus on the psychological theory, the design of experiments, and the interpretation of results instead of programming, debugging, and testing fitting routines. However, flexible and user-friendly software is not yet available to analyze MPT models with continuous hierarchical distributions.

To fit hierarchical MPT models, it is currently necessary either to implement an estimation routine from scratch (Klauer, 2010; Smith & Batchelder, 2010) or to build on model code for the software WinBUGS (Matzke, Dolan, Batchelder, & Wagenmakers, 2015; Smith & Batchelder, 2010). However, both of these previous hierarchical implementations are tailored to a specific MPT model (i.e., the pair-clustering model; Batchelder & Riefer, 1986) and require substantial knowledge and programming skills to fit, test, summarize, and plot the results of a hierarchical MPT analysis. Moreover, substantial parts of the analysis need to be adapted anew for each MPT model, which requires considerable effort and time and is prone to errors relative to relying on tested and standardized software.

As a remedy, we provide an accessible introduction to hierarchical MPT modeling and present the user-friendly and flexible software TreeBUGS to facilitate analyses within the statistical programming language R (R Core Team, 2016). Besides fitting models, TreeBUGS also includes tests for homogeneity of participants and/or items (Smith & Batchelder, 2008), posterior predictive checks to assess model fit (Gelman & Rubin, 1992; Klauer, 2010), within- and between-subjects comparisons, and MPT-tailored summaries and plots. TreeBUGS also provides novel statistical extensions that allow including both continuous and discrete predictors for the individual parameters.

In the following, we shortly describe the statistical class of MPT models and two hierarchical extensions: the beta-MPT and the latent-trait approach. We introduce the extensive functionality of TreeBUGS using the two-high threshold model of source monitoring (Bayen, Murnane, & Erdfelder, 1996). The online supplementary material (available at the Open Science Framework: https://osf.io/s82bw/) contains complete data and R code to reproduce our results. Note that we focus on an accessible introduction to hierarchical MPT modeling using TreeBUGS and refer the reader to Klauer (2010), Matzke et al. (2015), and Smith and Batchelder (2010) for mathematical details.

## Multinomial processing tree models

### Example: the two-high-threshold model of source monitoring (2HSTM)

Before describing the statistical details of the MPT model class in general, we introduce the source-monitoring model, which serves as a running example. In a typical source-monitoring experiment (Johnson, Hashtroudi, & Lindsay, 1993), participants first learn a list of items that are presented by two different sources. After study, they are asked whether the test items were presented by one of the sources (*Source A* or *Source B*) or whether they were not presented in the learning list (*New*). The substantive interest lies in disentangling recognition memory for the item from memory for the source while taking response and guessing tendencies into account.

**Fig. 1:**
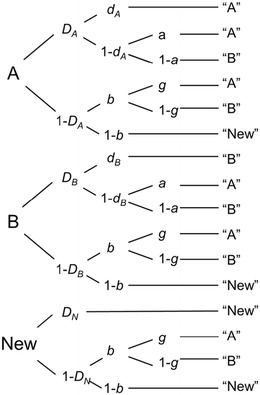
Two-high-threshold model of source monitoring (2HTSM). Participants are presented with learned items by two sources *A* and *B* along with new items, and they have to judge each item as belonging to either *Source A* or *Source B*, or being *New. D*_*A*_ = probability of detecting that an item presented by Source A is old; *D*_*B*_ = probability of detecting that an item presented by Source B is old; *D*_*N*_ = probability of detecting that an item is new; *d*_*A*_ = probability of correctly remembering that an item was presented by Source A; *d*_*B*_ = probability of correctly remembering that an item was presented by Source B; *a* = probability of guessing that an item that has been recognized as old is from Source A; *g* = probability of guessing that an item is from Source A if it was not recognized as old; *b* = probability of guessing that an item is old. Adapted from “Source Discrimination, Item Detection, and Multinomial Models of Source Monitoring,” by U. J. Bayen, K. Murnane, and E. Erdfelder, 1996, *Journal of Experimental Psychology: Learning, Memory, and Cognition*, *22*, p. 202. Copyright 1996 by the American Psychological Association

The two-high-threshold model of source monitoring (2HTSM; Bayen, et al., 1996), shown in Fig. 1, explicitly models these latent processes. Given an old item was presented by Source A, participants recognize it as old with probability *D*_*A*_, a parameter measuring item recognition memory. Conditionally on item recognition, the source memory parameter *d*_*A*_ gives the probability of correctly remembering the item’s source, which results in a correct response (i.e., A). If one of these two memory processes fails, participants are assumed to guess. If the test item itself is not recognized (with probability 1− *D*_*A*_), participants correctly guess that the item was old with probability *b*. Similarly, conditionally on guessing *old*, the parameter *g* gives the probability of guessing A. On the other hand, if the item is recognized with certainty as being *old* but the source is not remembered, participants only have to guess the source (with probability *a* for guessing A).

An identical structure of latent processes is assumed for items from Source B, using separate memory parameters *D*_*B*_ and *d*_*B*_. Regarding new items, detection directly results in a New response with probability *D*_*N*_, whereas the guessing probabilities are identical to those for the learned items. The expected probabilities for each of the nine possible response categories (three item types times three possible responses) are simply given by (a) multiplying the transition probabilities within each processing path in Fig. 1 (e.g., ${D}_A\cdotp {d}_A$ for answering “A” to a statement presented by Source A, due to recognition and source memory), and (b) summing these branch probabilities separately for each observable category [e.g., $P\left(``\mathrm{A}"\Big|\;\mathrm{Source}\;\mathrm{A}\right)={D}_A\cdotp {d}_A+{D}_A\cdotp \left(1-{d}_A\right)\cdotp a+\left(1-{D}_A\right)\cdotp b\cdotp g$]. To obtain the expected frequencies, the total number of responses per tree (e.g., the number of trials per item type) is multiplied by the expected probabilities. Note that the 2HTSM has eight parameters and only six free response categories, and is thus not identifiable. To render the model identifiable and obtain unique parameter estimates, we restricted some of the model parameters to be identical, on the basis of theoretical assumptions detailed below.

As an empirical example, we reanalyze data by Arnold, Bayen, Kuhlmann, and Vaterrodt (2013). Eighty-four participants had to learn statements that were presented by either a doctor or a lawyer (Source) and were either typical for doctors, typical for lawyers, or neutral (Expectancy). These two types of statements were completely crossed in a balanced way, resulting in a true contingency of zero between Source and Expectancy. Whereas the profession schemata were activated at the time of encoding for half of the participants (encoding condition), the other half were told about the professions of the sources just before the test (retrieval condition). Overall, this resulted in a 2 (Source; within subjects) × 3 (Expectancy; within subjects) × 2 (Time of Schema Activation; between subjects) mixed factorial design. After the test, participants were asked to judge the contingency between item type and source (perceived contingency *pc*). On the basis of the latent-trait approach, we (a) first analyze data from the retrieval condition; (b) show how to check for convergence and model fit, and perform within-subjects comparisons; (c) compare the parameter estimates to those from the beta-MPT approach; (d) include perceived contingency as a continuous predictor for the source-guessing parameter *a*; and (e) discuss two approaches for modeling a between-subjects factor (i.e., Time of Schema Activation).

### Likelihood function for the MPT model class

As is implied by their name, MPT models assume a product-multinomial distribution on a set of $K\ge 2$ mutually exclusive categories $\boldsymbol{C}=\left\{{C}_1,\dots, {C}_K\right\}$ (Batchelder & Riefer, 1999). The expected category probabilities of this product-multinomial distribution are given by nonlinear functions (i.e., polynomials) of the parameters, which are defined as unconditional or conditional transition probabilities of entering the latent cognitive states (Hu & Batchelder, 1994). The parameters are collected in a vector $\boldsymbol{\theta} =\left({\uptheta}_1,\dots, {\uptheta}_S\right)$, where each of the *S* functionally independent components is a probability with values in [0, 1].

Given a parameter vector ***θ***, the expected probability for a branch *B*_*ik*_ (i.e., the *i*th branch that terminates in category *C*_*k*_) is given by the product of the transition probabilities,

1 $$ P\left({B}_{ik}\Big|\boldsymbol{\uptheta} \right)={c}_{ik}{\displaystyle {\prod}_{s=1}^S}{\uptheta}_s^{a_{ik s}}{\left(1-{\uptheta}_s\right)}^{b_{ik s}}, $$

where ${a}_{iks}$ and ${b}_{iks}$ count the occurrences of the parameters ${\theta}_s$ and ($1-{\theta}_s$) in the branch *B*_*ik*_, respectively, and *c*_*ik*_ is the product of all constant parameters in this branch.

Assuming independent branches, the expected probability for a category *C*_*k*_ is then given by sum of the *I*_*k*_ branch probabilities terminating in this category,

2 $$ P\left({C}_k\Big|\boldsymbol{\uptheta} \right)={\displaystyle {\sum}_{i=1}^{I_k}{c}_{i k}}{\displaystyle {\prod}_{s=1}^S}{\uptheta}_s^{a_{i k s}}{\left(1-{\uptheta}_s\right)}^{b_{i k s}}. $$

The model’s likelihood is obtained by plugging these category probabilities into the density function of the product-multinomial distribution. For parameter estimation, this likelihood function is maximized either by general-purpose optimization methods (e.g., gradient descent) or by means of an MPT-tailored expectation-maximization algorithm (Hu & Batchelder, 1994; Moshagen, 2010), later improved by You, Hu, and Qi (2011).

### Hierarchical MPT models

As we outlined above, a violation of the i.i.d. assumption can result in biased parameter estimates. More specifically, heterogeneity can result in an underestimation or an overestimation of the standard errors for the parameter estimates and thus in confidence intervals that are too narrow or too wide, respectively (Klauer, 2006). Moreover, goodness-of-fit tests might reject a model based on aggregated data even though the model holds on the individual level. Smith and Batchelder (2008) showed that—even for a relatively homogeneous group of participants—the assumption of homogeneity of participants was violated whereas items in middle serial positions were homogeneous. Most importantly, participant heterogeneity is at the core of research questions that aim at explaining individual differences and thus require the estimation of individual parameters (e.g., in cognitive psychometrics; Riefer, Knapp, Batchelder, Bamber, & Manifold, 2002).

To address these issues, Bayesian hierarchical models explicitly account for the heterogeneity of participants (Lee, 2011). Essentially, hierarchical MPT models assume that the individual response frequencies follow the same MPT likelihood function as derived in the previous section, but with a separate parameter vector ***θ***_***p***_ for each participant *p*. Instead of estimating a single set of parameters for all participants (often called “complete pooling”) or assuming independent sets of parameters per participant (“no pooling”), individual parameters are modeled as random effects. According to this idea, the individual parameters are treated as random variables that follow some well-specified hierarchical distribution (in the present case, a transformed multivariate normal distribution or independent beta distributions). Importantly, this approach combines information from the individual and the group level (“partial pooling”) and thereby provides more robust parameter estimates than does fitting data for each participant separately (Rouder & Lu, 2005), because the collective error of the hierarchical estimates is expected to be smaller than the sum of the errors from individual parameter estimation.

The two hierarchical MPT approaches we consider here differ with respect to the assumed continuous hierarchical distributions of the individual parameters. In the *latent-trait* approach, the probit-transformed individual parameters are assumed to follow a multivariate normal distribution. In contrast, the *beta-MPT* assumes that individual parameters follow independent beta distributions.

Hierarchical models often rely on Bayesian inference with a focus on the posterior distribution of the parameters (Lee & Wagenmakers, 2014). Given the likelihood function of a model and some prior beliefs about the parameters, the posterior distribution describes the updated knowledge about the parameters after consideration of the data. Since analytical solutions and summary statistics of the posterior distribution (e.g., posterior means for each parameter) are often not available analytically, Bayesian inference employs Markov-chain Monte Carlo (MCMC) methods to draw samples from the posterior distribution. Based on a sufficient number of posterior samples, summary statistics such as the mean, the median, or quantiles can be easily computed to obtain parameter estimates, credibility intervals, and goodness-of-fit statistics.

### Beta-MPT approach

The beta-MPT approach (Smith & Batchelder, 2010) assumes that the individual parameters of participants are drawn from independent beta distributions. The beta distribution has a positive density on the interval [0, 1], which is the range of possible values for MPT parameters (i.e., probabilities). The density of the beta distribution for the *s*th MPT parameter ${\theta}_{ps}$ of person *p* depends on two positive parameters ${\alpha}_s$ and *β*_*s*_ that determine the shape of the distribution,

3 $$ g\left({\uptheta}_{ps}\Big|{\upalpha}_s,{\upbeta}_s\right)=\frac{\Gamma \left({\upalpha}_s+{\upbeta}_s\right)}{\Gamma \left({\upalpha}_s\right)\Gamma \left({\upbeta}_s\right)}{\uptheta}_{ps}^{\upalpha_s-1}{\left(1-{\uptheta}_{ps}\right)}^{\upbeta_s-1}, $$

where Γ(*x*) is the gamma function, which ensures that the density integrates to one.

**Fig. 2:**
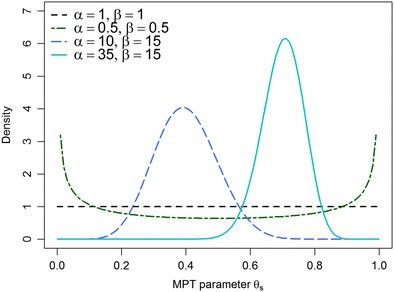
Density functions of the beta distribution for different shape parameters *α* and *β*

Figure 2 shows that the beta distribution covers a wide range of shapes to model individual differences in MPT parameters. If *α* or *β* is greater than one, the distribution is unimodal; if both parameters are equal to one, it is uniform; if both are smaller than one, the distribution is u-shaped; and if *α* > 1 and *β* < 1 (or vice versa), the distribution is monotonically increasing (or decreasing). To obtain summaries for the location and spread of the MPT parameters on the group level, the mean and variance of the beta distribution are computed as

4 $$ \mathrm{E}\left({\uptheta}_s\right)=\frac{\upalpha_s}{\upalpha_s+{\upbeta}_s} $$

and

5 $$ \mathrm{V}\mathrm{a}\mathrm{r}\left({\uptheta}_s\right)=\frac{\upalpha_s{\upbeta}_s}{\left({\upalpha}_s+{\upbeta}_s+1\right){\left({\alpha}_s+{\upbeta}_s\right)}^2}. $$

Note that the hierarchical distribution of the beta-MPT assumes independent MPT parameters across participants. Even though it is possible to estimate the correlation of parameters on the basis of posterior samples, the validity of the results is questionable, since it is not clear how influential the prior of independent parameters is. In extreme cases, the prior that the individual MPT parameters are independent may be so informative that very large sample sizes are required in order to obtain correlated posterior samples.

### Latent-trait approach

Cognitive abilities not only vary on an absolute level between participants, but also are often correlated (Matzke et al., 2015). For instance, two parameters that reflect different aspects of memory retrieval are likely to be similar within participants. For both statistical and substantive reasons, it might therefore be important to include parameter correlations in the hierarchical model explicitly. In the latent-trait model (Klauer, 2010), this is achieved by assuming that the transformed, individual parameter vector ${\uppi}^{-1}\left({\boldsymbol{\uptheta}}_{\boldsymbol{p}}\right)$ of a person *p* follows a multivariate normal distribution with group mean **μ** and a variance–covariance matrix **Σ**. The correlations between parameters are modeled explicitly by assuming a multivariate prior for the full vector of parameters ${\boldsymbol{\uptheta}}_{\boldsymbol{p}}$ (instead of using independent univariate priors for each vector components ${\theta}_{ps}$ as in the beta-MPT). The probit transformation ${\pi}^{-1}\left({\boldsymbol{\uptheta}}_{\boldsymbol{p}}\right)$ is defined component-wise by the inverse of the standard-normal cumulative density Φ and monotonically maps an MPT parameter ${\theta}_{ps}$ from the range (0, 1) to the real line. This is necessary in order to ensure that the transformed MPT parameters match the possible realizations of the normal distribution.

The model can equivalently be formulated as an additive decomposition of the probit-transformed parameters into a group mean **μ** and a participant random effect **δ**_***p***_ that follows a centered multivariate normal distribution (Matzke et al., 2015),

6 $$ {\varPhi}^{-1}\left({\boldsymbol{\uptheta}}_{\boldsymbol{p}}\right)=\boldsymbol{\upmu} +{\boldsymbol{\updelta}}_{\boldsymbol{p}}. $$

Note that this structure is similar to standard linear multilevel models with random intercepts for participants (Pinheiro & Bates, 2000) and will provide the starting point for including continuous predictors and discrete factors, as we describe below.

## TreeBUGS

TreeBUGS requires the statistical programing language R (R Core Team, 2016), the MCMC sampler JAGS (Plummer, 2003), and the R package runjags (Denwood, 2016). All programs are open-source software and available for free. The integration of TreeBUGS within R facilitates the overall work flow by enabling data preparation, analysis, plotting, and summarizing the results within a single programing environment. Moreover, the data generation and fitting functions of TreeBUGS can easily be wrapped into loops to run Monte Carlo simulations—for instance, to assess the precision of the parameter estimates for a given sample size.

However, for users less familiar with R, TreeBUGS also allows to import data, specify models, and export results using simple text files, which reduces the use of R to a few functions for model fitting only. Complete R code that serves as a user-friendly introduction for TreeBUGS is provided in the supplementary material (https://osf.io/s82bw).

TreeBUGS and the documentation are available via CRAN (https://CRAN.R-project.org/package=TreeBUGS) and can be installed by typing install.packages("TreeBUGS") into the R console.^1^ Once the package is installed, it needs to be loaded in each session via library(TreeBUGS).

Note that TreeBUGS searches for data, model, and restriction files within the current working directory, which needs to be adjusted to the correct path once (e.g., using the command setwd("C:/mpt/")).

### Format of models, restrictions, and data

To specify an MPT model, TreeBUGS requires a text file in the .eqn standard, which is also used by other software such as multiTree (Moshagen, 2010). The first line of the model file is ignored by TreeBUGS and reserved for comments (similarly to multiTree).^2^ Each of the remaining lines defines a single branch probability of the MPT model and includes three entries separated by white space: the tree label, the category label, and the branch equation. For instance, the first lines of the .eqn-file for the 2HTSM (i.e., model/2htsm.eqn in the Online Appendix) are
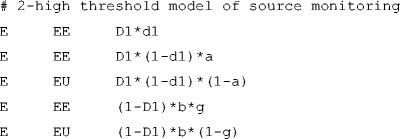
where *E* describes schematically the expected sources (e.g., medical statements presented by a doctor), *U* describes schematically unexpected sources (e.g., medical statements presented by a lawyer), and *N* describes new items not previously learned.

Often, some of the MPT parameters are constrained to be identical or constant based on theoretical reasons or to ensure the identifiability of the model. Within TreeBUGS, such constraints are added by a list and may contain equality constraints and constants,

restrictions = list("D1 = D2 = D3", "d1 = d2", "a = g")

Alternatively, one can specify the path to a text file that includes one constraint per row. In the present example (included in model/restrictions.txt), we assume that the probability of remembering a learned item is identical for both sources and also identical to the probability of recognizing an item as *New* (i.e., ${D}_A={D}_B={D}_N$). Similarly, source memory is assumed to be equal for the two sources (${d}_A={d}_B$), and source guessing is assumed to be independent of whether participants recognized the item ($a= g$).

To fit a hierarchical model, TreeBUGS requires a table of individual frequencies with participants in rows and observed categories in columns. These data can either be provided in a comma-separated .csv file (with category labels in the first row) or as a matrix or data frame within R (with column names matching the observable categories in the model file). For our example, the first lines of the data file data/data_retrieval.csv, which provides the response frequencies in the retrieval condition, are
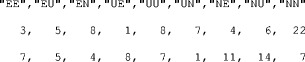


### Testing the homogeneity of participants

Before fitting a hierarchical model to individual data instead of fitting a standard MPT model to aggregated data, it is important to check whether participants are actually heterogeneous (Smith & Batchelder, 2008). If this test does not reject the null hypothesis that individual frequencies are identically distributed, the simpler standard MPT model should be used, since it reduces the possibility of overfitting (Smith & Batchelder, 2008).^3^

To test for heterogeneity among participants or items, TreeBUGS implements the asymptotic *χ* ^2^ test and the permutation test proposed by Smith and Batchelder (2008). The former allows testing for participant heterogeneity under the assumption that items are homogeneous and requires the same table of individual frequencies as described in the previous section. In our example, the χ^2^ test for participant heterogeneity is run as
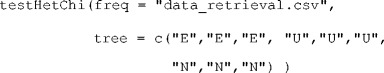


The argument tree indicates which columns of the frequency table freq belong to separate multinomial distributions (here, the nine observed categories belong to the three trees of the 2HTSM). As is indicated by the small *p* value [*χ* ^2^(138) = 325.1, *p* = 3.9 · 10^−17^], there is substantial heterogeneity between participants.

In contrast to the *χ* ^2^ test, the permutation test allows to test person homogeneity even if items are heterogeneous. To do so, the data need to be provided in the long format with the participant code in the first column, the item label or number in the second column, and the observed response in the third column. Using 10,000 permutations, we can run this test via
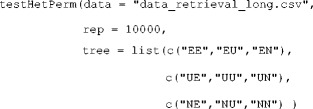


**Fig. 3:**
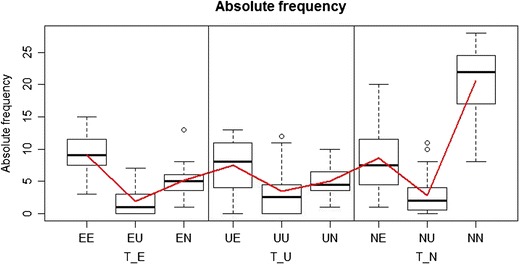
Plot of the observed frequencies using the function plotFreq("data_retrieval.csv"). Boxplots show the distributions of individual frequencies per response category and MPT tree, whereas the solid red line shows the mean frequencies

In contrast to the *χ* ^2^ test, the argument tree is now a list in which the elements are vectors with category labels for each multinomial distribution (i.e., for each MPT tree). In our example, this test also indicates a significant deviance from the null hypothesis that persons are homogeneous (*p* < .001). Moreover, TreeBUGS also provides a graphical assessment of participant heterogeneity by plotting the individual against the mean (absolute or relative) frequencies via the function plotFreq, illustrated in Fig. 3.

### Fitting a latent-trait MPT model

In the simplest scenario, the user only needs to specify the paths to the files with the model equations, the parameter restrictions, and the individual frequencies to fit a latent-trait MPT model,

fittedModel <− traitMPT(eqnfile="eqnfile.eqn", data="data.csv", restrictions="restrictions.txt")

However, this approach relies on several defaults regarding the hyperpriors on the group-level parameters **μ** and **Σ** and details about the MCMC sampling scheme. We strongly advice the user to adjust these defaults depending on theoretical considerations and on the convergence of a model, respectively. Based on the .eqn model file and the restrictions, TreeBUGS creates a JAGS file that is then used to obtain MCMC samples. By default, this file is only saved temporarily, but it can be saved to the working directory for a closer inspection using the argument modelfilename="2htsm.jags”. This file can also be used when working with JAGS directly.

By default, TreeBUGS samples 20,000 iterations of the MCMC sampler of which the first 2,000 iterations are dropped to avoid dependencies on the starting points (the so-called *burn-in period*). More complex MPT models might require more iterations to achieve convergence of the MCMC sampler and thus an adjustment of n.iter and n.burnin to sample more iterations and remove more burn-in samples, respectively. To reduce the load on the computer’s memory, TreeBUGS only retains every fifth iteration of the MCMC samples to compute summary statistics. In the case of highly auto-correlated MCMC samples, this so-called thinning results only in a minor loss of information since the dropped samples are very similar to the retained ones. The user can change the thinning rate using n.thin.

By default, TreeBUGS obtains posterior samples from three MCMC chains in parallel using different starting values (n.chains=3). The sampling from multiple MCMC chains allows checking convergence by assessing whether the discrepancy between chains is sufficiently small. Note that TreeBUGS offers the option autojags to run JAGS until some convergence criterion is reached, for instance, until the variance of parameters between chains is sufficiently small (Gelman & Rubin, 1992). However, note that this might require substantial computing time. Convergence issues can also be due to nonidentifiable MPT parameters; this highlights the importance of checking the identifiability of a model using either numerical (Moshagen, 2010) or analytical (Schmittmann, Dolan, Raijmakers, & Batchelder, 2010) methods.

After fitting the model, TreeBUGS returns an object that includes the MCMC samples and summary statistics tailored to MPT models. By default, the output is only saved temporarily within R. Alternatively, TreeBUGS allows to export summary statistics of the posterior to a text file (e.g., parEstFile = "results.txt") or the fitted model with all posterior samples to an R data file (e.g., posteriorFile = "posterior.RData").

Often, one is interested in differences, ratios, or other function of the core MPT parameters based on the posterior distribution. To test such transformations on a within-subjects level, TreeBUGS provides the argument transformedParameters = list ("deltaDd = D1-d1"), which computes the difference in memory parameters using the group-mean posterior samples (see below for corresponding individual-level and between-subjects analyses).

When combining all of these arguments, a possible call to TreeBUGS could be
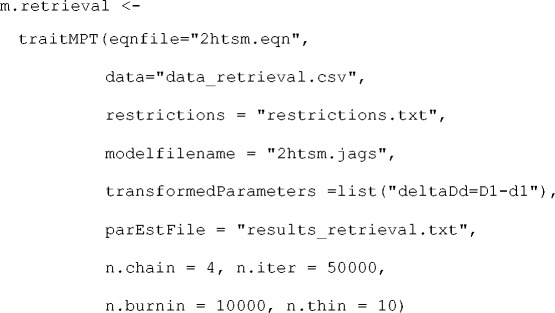


On a notebook with an Intel i5-3320M processing unit, drawing posterior samples for this model requires approximately two minutes. In the following, we refer to this fitted model when showing plots and summaries of empirical results.

### Monitoring convergence

**Fig. 4:**
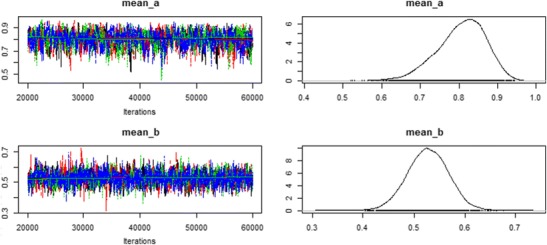
Visual check of convergence using the function plot(m.retrieval, parameter = "mean")

As we mentioned above, it is important to ensure that the posterior distribution is approximated sufficiently well when relying on MCMC sampling (Gelman & Hill, 2007). Mathematical proofs only ensure that the MCMC sampler approximates the posterior as the number of iterations goes to infinity, but this approximation might be insufficient and biased for finite numbers of iterations. Therefore, it is important to check a model’s convergence graphically—for instance, by using autocorrelation or time series plots. TreeBUGS provides these plots tailored to the MPT parameters of interest based on the R package coda (Plummer, Best, Cowles, & Vines, 2006). For instance, a time-series and density plot of the group-mean parameters is obtained by typing plot(m.retrieval, parameter = "mean"),resulting in the plot in Fig. 4, which indicates good convergence (i.e., the MCMC chains look like “fat, hairy caterpillars”). To obtain autocorrelation plots for the MCMC samples, it is sufficient to add the argument type = "acf".

Besides these graphical tests, the summary output of TreeBUGS provides an estimate for the effective sample size (i.e., the estimated number of iterations corrected for autocorrelation) and the convergence statistic $\widehat{R}$ for each parameter, which quantifies the ratio of between-chain and within-chain variance and should be close to one (e.g., $\widehat{R}<1.05$; Gelman & Rubin, 1992). If there are any indications that the model has not converged, it is necessary to fit the model using more iterations. To reuse posterior samples and save computing time, TreeBUGS allows retaining previously sampled posterior values using the function extendMPT.

### Priors on the group-level parameters

To fit the latent-trait model, prior distributions are required on the group-level parameters **μ** and **Σ**. The defaults of TreeBUGS use weakly informative priors following the proposals of Klauer (2010) and Matzke et al. (2015). The priors for the group means μ_*s*_ are standard normal distributions that imply uniform distributions on the group means in probability space (Rouder & Lu, 2005). Regarding the covariance matrix **Σ**, a scaled inverse Wishart prior is used, similar as in many other hierarchical models (Gelman & Hill, 2007). A weakly informative parameterization of the inverse Wishart prior is given by an identity scale matrix of size $S\times S$ with $S+1$ degrees of freedom. Since the standard inverse Wishart prior informs the parameter variances to a substantial degree, the standard deviations of the parameter are multiplied by the scaling parameters ξ_*s*_ to obtain a less informative prior (for details, see Klauer, 2010). Moreover, the scaling parameters often improve convergence of the MCMC sampler (Gelman & Hill, 2007). For the scaling parameters ξ_*s*_, TreeBUGS assumes a uniform distribution on the interval [0, 10] by default.

In certain scenarios, it might be desirable to change these default priors for the group-level parameters—for instance, in order to perform prior sensitivity analyses, to implement theoretically informed priors (Vanpaemel, 2010), or to adjust the priors to account for reparameterized order constraints (Heck & Wagenmakers, 2016). For these purposes, TreeBUGS allows the user to modify the default priors for the group-level parameters. Regarding the covariance matrix **Σ**, TreeBUGS allows the user to change the scale matrix and the degrees of freedom of the inverse Wishart prior using the arguments V and df, respectively, and the prior for the scale parameters **ξ** by the argument xi.

As an example regarding the group means **μ**, more-informative priors might be placed on the guessing parameters if the guessing rates are theoretically predicted to be around .50 for all participants (Vanpaemel, 2010). To implement this idea, one can change the priors on the latent, probit-scaled group means by adding to the call:
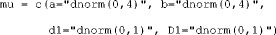


Note that the input is directly passed to JAGS, which parameterizes the normal distribution dnorm by the mean and the precision (i.e., the inverse of the variance, $\tau =1/{\sigma}^2$). Accordingly, the term "dnorm(0,4)" defines slightly more precise priors for *a* and *b* on the probit scale (i.e., normal distributions with mean zero and standard deviation 0.5, implying a mean of .50 on the probability scale) than for the default, standard-normal priors for the group means of *d* and *D*. For a complete overview of possible distributions, we refer the reader to the JAGS manual (Plummer, 2003).

**Fig. 5:**
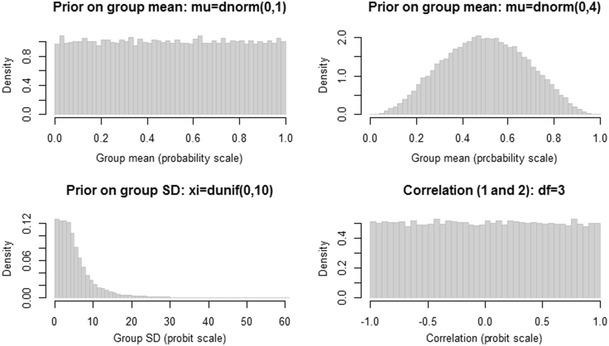
Prior distributions for the MPT group-level parameters of the latent-trait MPT model

To get an intuition about the effects of different priors on the parameter means, *SD*s, and correlations, TreeBUGS provides a function that draws samples and plots histograms for a given set of priors:
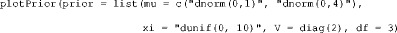


This example defines separate priors for the latent means μ_*s*_ of two MPT parameters (i.e., the standard-normal and the more precise prior "dnorm(0,4)" discussed above). The remaining arguments represent the default priors of TreeBUGS for the latent-trait MPT—that is, a uniform distribution on the interval [0, 10] for the scaling parameters ξ_*s*_ (the argument xi is used for both parameters) and the Wishart prior with scale-matrix *V* (the two-dimensional identity matrix diag(2)) and three degrees of freedom. Figure 5 shows that these priors imply a uniform prior and a more-informative, centered prior for the inverse-probit-transformed means $\pi \left(\upmu \right)$, respectively. Moreover, the prior distribution on the latent-probit *SD* is weakly informative, whereas the prior on the correlation is uniform. Note, however, that the use of informative priors is still a controversial topic (e.g., Wagenmakers, 2007). In any case, the possibility to change priors in TreeBUGS allows researchers to run prior sensitivity analyses by repeatedly fitting the same model with different priors.

### Assessing goodness of fit

Before interpreting the parameters of an MPT model, it is necessary to check whether the model actually fits the data. Within the maximum-likelihood framework, researchers usually rely on the likelihood-ratio statistic *G* ^2^ to test goodness of fit, which quantifies the discrepancy between observed and expected frequencies and is asymptotically *χ* ^2^ distributed with known degrees of freedom (Read & Cressie, 1988; Riefer & Batchelder, 1988). For hierarchical Bayesian models, conceptually similar methods exist to compare the observed frequencies against the frequencies predicted by the model’s posterior.

**Fig. 6:**
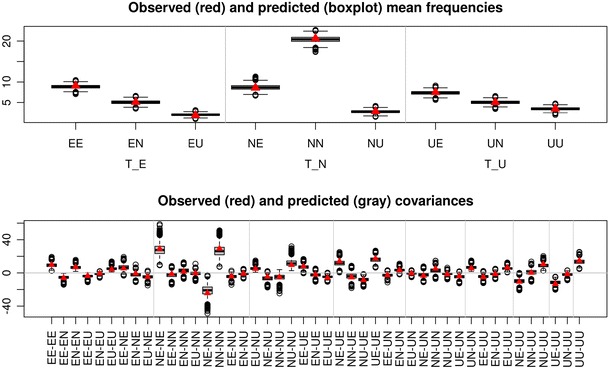
To assess model fit, the function plotFit shows the observed (red triangles) against the posterior-predicted (box plots) data in terms of (top) mean frequencies and (bottom) covariances

These posterior predictive checks can be performed graphically by plotting the observed mean frequencies against the distribution of mean frequencies that are sampled from the hierarchical model, using the posterior samples as data-generating parameters. Within TreeBUGS, such a plot of mean frequencies is obtained by plotFit(fittedModel). Similarly, the observed covariance of the individual frequencies can be plotted against that of the posterior predicted frequencies by adding the argument stat = "cov". Figure 6 shows the resulting plots, which indicate a satisfactory model fit because the observed and predicted values differ only slightly.

A quantitative assessment of model fit is provided by posterior predictive tests (Meng, 1994). On the basis of the posterior samples, these tests rely on a statistic that quantifies the discrepancy between the expected data (conditional on the posterior) and the observed (*T*_obs_) and the posterior-predicted data (*T*_pred_), respectively. Based on these two distributions of the test statistic, the proportion of samples is computed for which *T*_obs_ < *T*_pred_, the so-called posterior predictive *p* value (PPP). Whereas small PPP values close to zero indicate insufficient model fit, larger values indicate satisfactory model fit (e.g., PPP > .05). Note, however, that the exact distribution of these PPP values for the case that the model generated the data is not uniform contrary to goodness-of-fit *p* values in a frequentist framework (Meng, 1994).

For hierarchical MPT models, Klauer (2010) proposed the test statistics *T*_1_ and *T*_2_, which focus on the mean and covariance of the individual frequencies, respectively. The *T*_1_ statistic computes distance between observed (predicted) and expected mean frequencies using the formula for Pearson’s *χ* ^2^ statistic. Similarly, *T*_2_ computes the summed differences between observed (predicted) and expected covariances, standardized by the expected standard deviations. Using the individual-level MPT parameters, TreeBUGS computes both test statistics either directly when fitting a model by adding the argument ppp = 1000 to the function traitMPT (which resamples 1,000 posterior samples) or by calling the function PPP(fittedModel, M = 1000) separately after model fitting. Besides the PPP values for *T*_1_ and *T*_2_, testing the mean frequencies and covariances, respectively, the output also provides PPP values for all participants separately by applying the *T*_1_ statistic to individual response frequencies. Note that the underlying TreeBUGS function posteriorPredictive draws posterior-predictive samples using either the participant- or group-level parameters, which facilitates the computation of any other test statistic of interest.

Besides these tests for absolute goodness of fit, TreeBUGS also allows to compute the deviance information criterion (DIC) to select between competing models (Spiegelhalter, Best, Carlin, & van der Linde, 2002) by adding the argument dic=TRUE. Similar to the AIC or BIC information criteria, the DIC trades off model fit and model complexity. After fitting each of the competing hierarchical MPT models, the model with the smallest DIC value performs best in this trade-off. Note, however, that the DIC has been criticized for being “not fully Bayesian” and having undesirable properties (e.g., Gelman, Hwang, & Vehtari, 2014; Plummer, 2008; Vehtari & Ojanen, 2012).

### Summarizing and plotting results

There are several convenient ways to summarize and visualize the posterior distribution of the parameters. A full summary, including group-parameter estimates, transformed parameters, posterior predictive checks, and DIC (if any of these were computed), is provided either by summary(fittedModel) or in the output file specified via parEstFile, as described above. Note that individual parameter estimates are provided by default only in the latter case. Within R, the function

getParam(m.retrieval, parameter = "theta", stat = "summary")

allows the user to extract individual parameter estimates in R (as well as estimated group means and correlations) for a closer inspection and further processing.

**Fig. 7:**
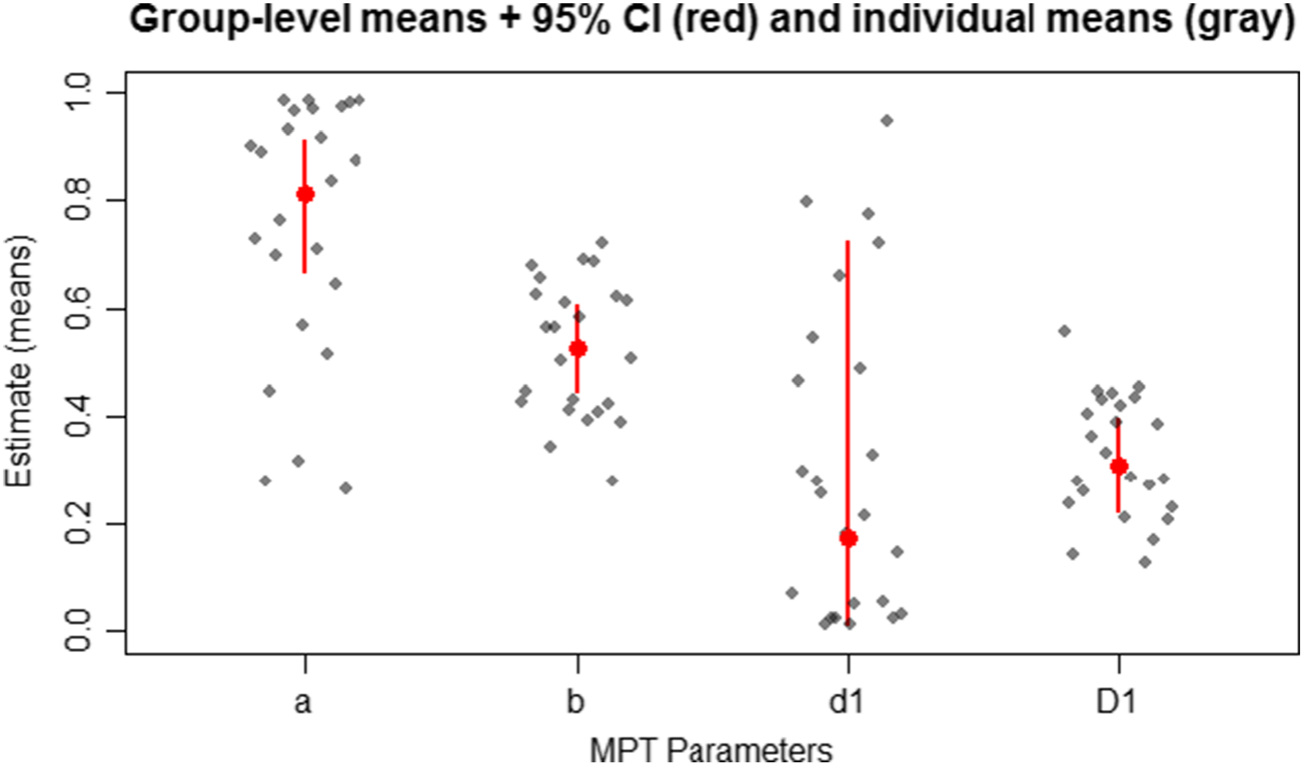
The function plotParam shows the posterior-mean estimates of the individual and mean parameters (including 95% credibility intervals for the latter)

**Fig. 8:**
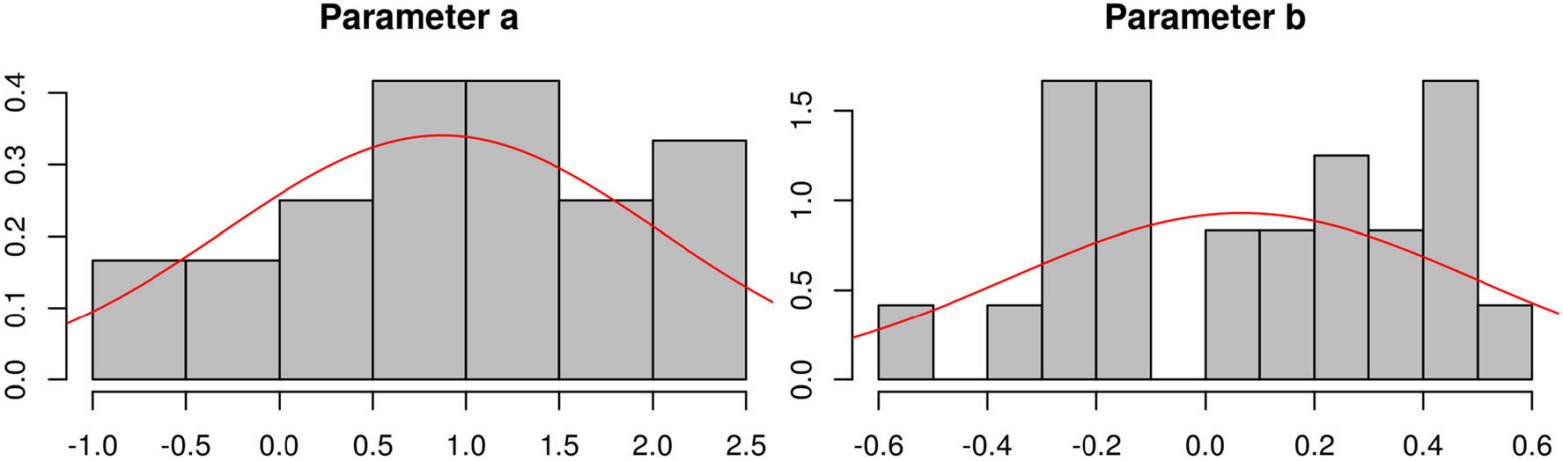
The function plotDistribution compares the distributions of individual posterior-mean estimates (gray histograms) against the group-level distributions assumed by the posterior means of the hierarchical latent-trait parameters (red density curves)

**Fig. 9:**
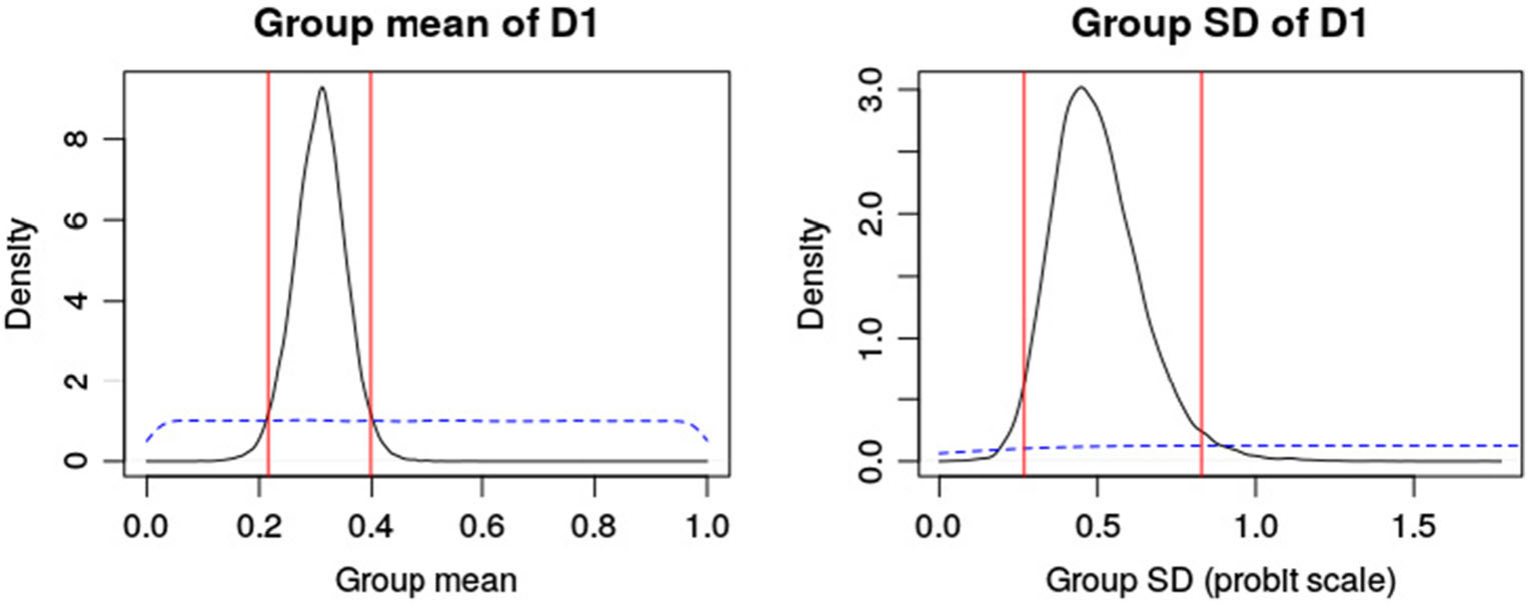
Plot of the prior distributions (dashed blue lines) versus the posterior distributions (solid black lines) of the group-level mean and *SD* of the MPT parameter *D*. The 95% credibility interval is shown by the pairs of vertical red lines

To summarize the results graphically, the function plotParam in Fig. 7 shows posterior-mean parameter estimates on the group level (including 95% Bayesian credibility intervals) and on the individual level (alternatively, the argument estimate="median" allows the user to plot posterior medians). For a closer inspection of the distribution of individual parameter estimates, Fig. 8 shows the output of the function plotDistribution, which compares histograms of the posterior means per participant with the expected density based on the estimated group-level parameters **μ** and **Σ** (on either the latent probit or the probability scale). To assess the amount of information provided by the data, plotPriorPost compares the prior densities of the parameters against the estimated posterior densities, as is shown in Fig. 9. If the posterior is markedly peaked as compared to the prior, the data are highly informative.

### Within-subjects comparisons

In psychological studies, participants often perform identical tasks in different experimental conditions. Such within-subjects factorial designs are often implemented in MPT models by using a separate set of parameters for each of the conditions. In an .eqn file, this requires the repetition of an MPT model structure with separate labels for trees, categories, and parameters per condition. To facilitate within-subjects comparisons, TreeBUGS therefore provides a function that replicates the MPT model equations multiple times with different labels per condition and returns the corresponding .eqn file. For instance, the call
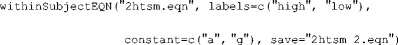
replicates the 2HTSM model equations for two memory strength conditions with invariant labels for the parameters *a* and g across conditions, but separate labels “high” and “low” for all trees, categories, and remaining parameters.

When fitting an MPT model, within-subjects comparisons can be tested with respect to the group-level mean parameters by using the argument transformedParameters, as shown above. Additionally, to perform tests on the individual parameters, transformations of parameters (e.g., differences or ratios) can be estimated after fitting a model using the function
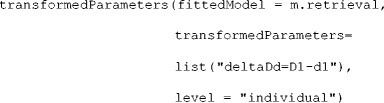
 which returns posterior samples for the differences in the memory parameters *D* and *d* for each participant.

### Between-subjects comparisons

If a factor is manipulated between subjects in an experiment, two or more separate hierarchical models can be fitted for the conditions similarly as shown above. Statistically, this implies that the participant random effects follow different hierarchical distributions across conditions. On the basis of these fitted models, the posterior samples can be used to compute differences, ratios, or other functions of the mean parameters **μ** between conditions to assess the effect of an experimental manipulation on the MPT parameters. Note that this procedure does not provide a strict hypothesis test for the difference in means, it rather allows to compute a credibility interval of the difference (Smith & Batchelder, 2010, p. 175).

In our empirical example, we can obtain an estimate for the difference in recognition memory between the retrieval and encoding conditions as measured by the parameter *D* by betweenSubjectMPT(m.retrieval, m.encoding, par1 = "D1"),where m.retrieval and m.encoding are the latent-trait MPT models fitted to the two conditions separately. By default, TreeBUGS computes (a) the difference in the mean parameters and (b) the proportion of samples for which ${\mu}_{\mathrm{Dr}}<{\mu}_{\mathrm{De}}$ (user-specified functions such as the ratio of parameters can be estimated by the argument stat="x/y"). TreeBUGS returns a summary that indicated no substantial effect in our example ($\varDelta \widehat{D}=.07$ with the 95% credibility interval $\left[-.03,.18\right]$; ${p}_B=.069$).

### Fitting a hierarchical beta MPT model

The TreeBUGS function betaMPT fits a hierarchical beta-MPT model (Smith & Batchelder, 2010) with mostly identical arguments as for traitMPT. The most important difference concerns the specification of the priors for the group-level parameters, that is, the priors for the shape parameters ***α*** and ***β*** of the hierarchical beta distributions. Similar to the component-wise priors on the group means **μ** in the latent-trait MPT, the defaults can be changed by the arguments alpha and beta either simultaneously for all MPT parameters (by using a single input argument) or separately for each MPT parameter (by using named vectors).

Regarding default priors, Smith and Batchelder (2010, p. 182) proposed relying on weakly informative priors on the shape parameters. Specifically, their WinBUGS code used the “zeros-trick,” which results in approximately uniform priors on the group-level mean and *SD* on the probability scale.^4^ This prior is available in the TreeBUGS function betaMPT via the arguments alpha="zero" or beta="zero", but it often causes JAGS to crash (similar as for WinBUGS; Smith & Batchelder, 2010, p. 182). Therefore, TreeBUGS uses a different default for the prior distribution on the shape parameters *α* and *β* (i.e., a gamma distribution with shape 1 and rate 0.1).

To compare different priors for the beta-MPT model, TreeBUGS plots the implied prior distributions for the group mean and standard deviation of the MPT parameters by
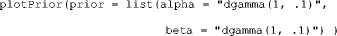


**Fig. 10:**
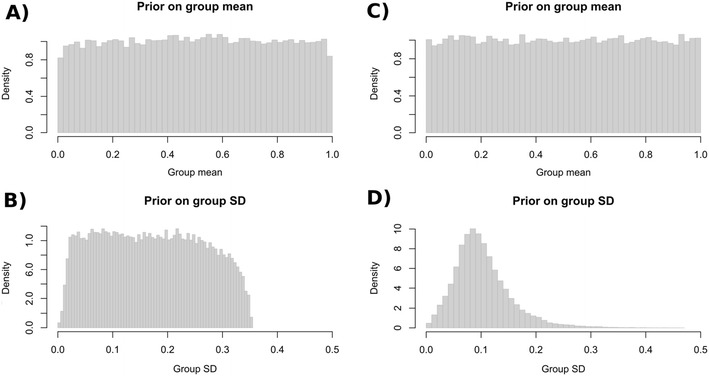
Implied prior distributions on the group means and *SD*s of individual MPT parameters based on the “zeros-trick” (panels A and B; Smith & Batchelder, 2010) and Gamma(1, 0.1) priors (panels C and D), for the parameters *α* and *β* of the hierarchical beta distribution

Figure 10 shows that both the “zeros-trick” and the gamma prior are uniform on the mean (panels A and C, respectively), whereas the former is less informative than the latter with respect to the group-level *SD* (panels B and D, respectively). However, the gamma prior used by default in TreeBUGS matches the theoretical expectation that individual MPT parameters actually differ (i.e., *SD*s close to zero are less likely) but are still similar to some degree (i.e., large *SD*s are less likely). Moreover, when choosing priors on a probability scale, it is important to consider that large *SD*s are only possible if the group-level mean is around $\overline{\theta}=.50$ (due to the constraint $S D\left(\theta \right)\le \sqrt{\overline{\theta}\left(1-\overline{\theta}\right)}$ ) and if the individual MPT parameters follow a uniform or even bimodal distribution (i.e., parameters are close to zero for some participants but close to one for others). To test whether different priors actually impact parameter estimation, or whether the data overwhelm the prior, TreeBUGS facilitates sensitivity analyses for a given model and sample size by changing the default prior.

In previous analyses, parameter estimates for the beta-MPT model were often similar to those for the latent-trait MPT model (e.g., Arnold, Bayen, & Smith, 2015). Table 1 shows the results of both analyses for the retrieval condition from Arnold et al. (2013). To facilitate the comparison, we transformed the probit mean μ and variance σ^2^ in the latent-trait MPT to the probability scale using the TreeBUGS function probitInverse. This function computes the implied mean and *SD* of individual MPT parameters on the probability scale given a normal distribution on the latent probit scale.^5^ Note that the resulting group-level mean differs from $\uppi \left(\upmu \right)$ (i.e., the inverse-probit transformed parameter μ) because the variance σ^2^ on the probit scale shifts the probability mean toward .50. However, we used the bivariate transformation probitInverse only for the present comparison with the beta-MPT model, but report $\uppi \left(\upmu \right)$ and σ in the remainder of the article (in line with most previous applications).

**Table 1:** Comparison of parameter estimates of a latent-trait MPT model and a beta-MPT model

Parameter	Latent-Trait MPT				Beta-MPT				Participant Estimates	
	Mean		*SD*		Mean		*SD*		Correlation	Mean Abs. Difference
*a*	.714	(.056)	.270	(.040)	.766	(.047)	.223	(.035)	.999	.011
*b*	.523	(.036)	.155	(.028)	.524	(.034)	.140	(.023)	.999	.005
*d*	.378	(.095)	.394	(.064)	.191	(.080)	.181	(.086)	.916	.166
*D*	.329	(.041)	.159	(.038)	.328	(.036)	.135	(.028)	.996	.011

Analysis of the retrieval condition of Arnold et al. (2013). For the group-level mean and *SD* parameters, posterior means (and SDs) are shown. Participant estimates refer to posterior means of the individual MPT parameters

Table 1 shows that the estimates based on the beta-MPT and the latent-trait MPT model were similar for the group-level means and *SD*s. Moreover, correlations and mean absolute differences between individual posterior-mean estimates across models were high. The largest discrepancy was observed for the parameter *d*, which is also the parameter estimated with most uncertainty. Moreover, the small sample size of *N* = 24 might have contributed to the diverging mean estimates. In line with general Bayesian principles, this illustrates that the type of prior distribution on the group level (beta vs. latent-trait) has a stronger impact on those parameters that are informed less by the data.

## Including covariates and predictors for MPT parameters

### Correlations of MPT parameters and covariates

When testing hypotheses regarding individual differences, substantive questions often concern the correlation of covariates such as age or test scores with the cognitive processes of interest as measured by the individual MPT parameters (Arnold, Bayen, & Böhm, 2015; Arnold et al., 2013; Michalkiewicz & Erdfelder, 2016). Hierarchical MPT models are ideally suited to assess such hypotheses, since they naturally provide separate parameter estimates per participant. Moreover, instead of computing a single correlation coefficient using fixed parameter estimates, the correlation of interest can be computed repeatedly for all posterior samples, which allows for quantifying the uncertainty due to parameter estimation. Importantly, however, this approach does not take the sampling error of the population correlation into account, which depends on the number of participants (Ly et al., in press). As a remedy, Ly et al. (in press) proposed to estimate the posterior distribution of the population correlation by (1) computing correlations for all posterior samples separately, (2) approximating the sampling-error-corrected posterior distribution of the population correlation for each replication (Ly, Marsman, & Wagenmakers, 2017), and (3) averaging over the resulting posterior densities.

TreeBUGS implements this method in two steps. First, the functions traitMPT and betaMPT compute correlations between MPT parameters and covariates if a data set with individual values on the external variables is provided. In the case of our empirical example, the sample correlation of age with the MPT parameters is estimated by adding the argument covData = "age_retrieval.csv"—that is, the path to a .csv file that includes the variable age. Besides external data files, TreeBUGS also accepts matrices or data frames via covData. In both cases, the order of participants must be identical to that of the data with individual frequencies. For both the latent-trait MPT and the beta-MPT, TreeBUGS computes the correlations of all continuous variables with the posterior values of the individual MPT parameters. If the argument corProbit = TRUE is added, correlations are instead computed using the individual parameters on the probit-transformed scale.

**Fig. 11:**
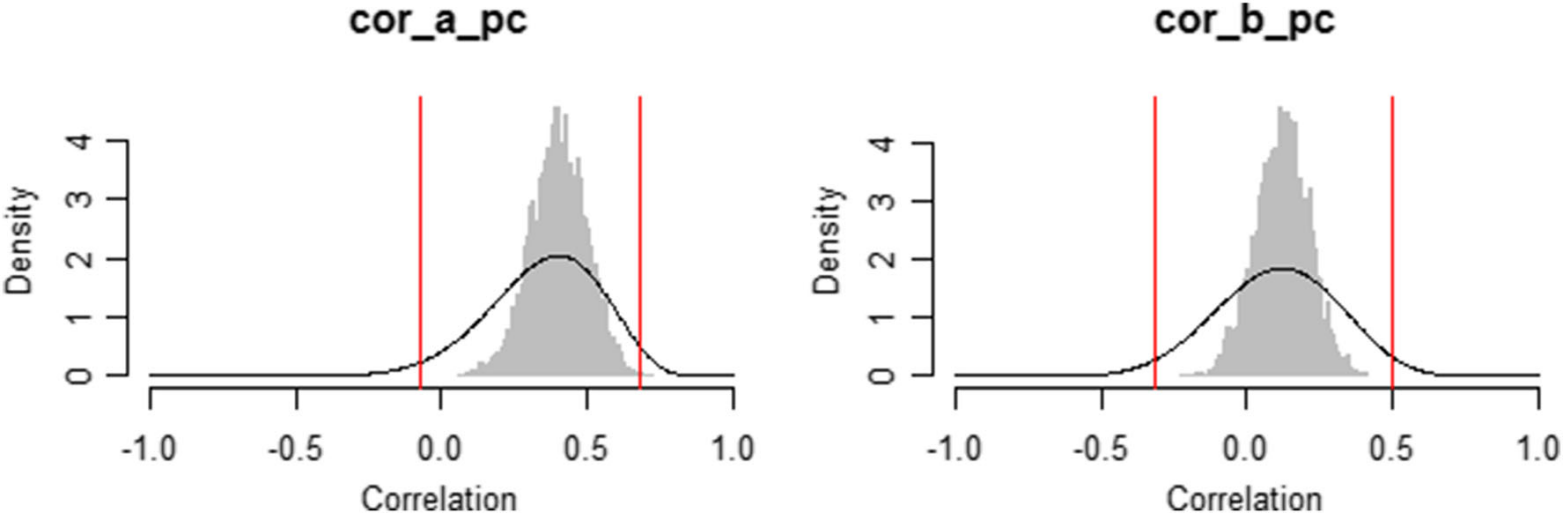
Comparison of the posterior distribution of the sample correlation, which only accounts for the uncertainty of the parameter estimates (gray histograms), with the posterior of the population correlation, which also accounts for the number of participants (black density curves, with 95% credibility intervals indicated by vertical red lines)

In a second step, the function correlationPosterior reuses these posterior samples of the sample correlation to estimate the population correlation, thereby accounting for the number of participants (Ly et al., in press). Besides mean estimates and credibility intervals, this function plots the posterior samples of the sample correlation (gray histograms) against the posterior distribution of the population correlation (black density curves, including 95% credibility intervals indicated by vertical lines). Figure 11 shows that the posterior of the population correlation is wider, which indicates the additional uncertainty due to sampling error.

### Continuous predictors for MPT parameters

In cognitive psychometrics, it might be of interest to test whether some variable affects the probability that a specific cognitive process occurs—that is, to regress the individual MPT parameters on external covariates. In our example, the probability-matching account predicts that the source-guessing parameter *a* is driven by the perceived source contingency (Arnold et al., 2013). To implement such a theoretical hypothesis statistically, we expanded the latent-trait MPT approach in Eq. 6 by a linear regression on the probit scale, as suggested by Klauer (2010, p. 92),

7 $$ {\varPhi}^{-1}\left({\uptheta}_{ps}\right)={\upmu}_s+{\mathbf{X}}_{\boldsymbol{s}}{\beta}_{\boldsymbol{s}}+{\updelta}_{ps}, $$

where **X**_***s***_ is a design matrix with covariates, and **β**_***s***_ a vector of regression coefficients for the *s*th MPT parameter (for a similar, frequentist approach, cf. Coolin, Erdfelder, Bernstein, Thornton, & Thornton, 2015).

Substantively, positive regression weights ${\beta}_{sk}$ imply a higher probability that the cognitive process *s* occurs as the covariate increases. Moreover, the inclusion of predictors is likely to result in a reduction of the variance of individual MPT parameters, and thus sheds light on possible sources of parameter variability.

Obviously, priors are required for the regression coefficients **β**_***s***_. Given that covariates can substantially differ in location and range, we assume scale-invariant default priors for the regression coefficients. Specifically, the columns of **X**_***s***_ are *z*-standardized to have a mean of zero and a variance of one. Based on this standardization, we assume weakly informative, multivariate Cauchy priors for each of the standardized regression coefficients **β**_***s***_ similar to the priors used by Rouder and Morey (2012) for standard linear regression modeling. For each MPT parameter *s*, this is achieved by independent univariate normal priors on the regression coefficients for the predictors $k=1,\ldots,{K}_s$,

8 $$ {\upbeta}_{s k}\sim \mathrm{Normal}\left(0,{g}_s\right), $$

and an inverse gamma prior on the variance *g*_*s*_,

9 $$ {g}_s\sim \mathrm{Inverse}\ \mathrm{Gamma}\left(1/2,{v}^2/2\right), $$

with a fixed scale parameter $v=1$ [which reduces Eq. 9 to an inverse χ^2^(1) prior].^6^ By defining a single variance parameter *g*_*s*_ for all slopes ${\beta}_{s1},\ldots,{\beta}_{s{ K}_s}$ of an MPT parameter *s*, a multivariate Cauchy prior is defined for **β**_***s***_ (Rouder & Morey, 2012). To change these defaults, other priors on *g*_*s*_ can be specified via the argument IVprec. For instance, different scale parameters *v* are specified by IVprec = "dgamma(1/2,v^2/2)" (with v being replaced by a fixed number), and standard-normal distributions on ${\beta}_{sk}$ are realized by IVprec = "dcat(1)" (which implies a fixed variance ${g}_s=1$).

Note that our default priors differ slightly from those proposed by Rouder and Morey (2012). First, TreeBUGS implements the multivariate Cauchy prior for multiple predictors of an MPT parameter under the additional assumption that the covariates are independent. Technically, this is due to the constraint that the predictors are normalized with respect only to their variance, but not to their covariance (cf. Rouder & Morey, 2012). Nevertheless, the default prior of TreeBUGS allows for parameter estimation of hierarchical MPT models, especially if the predictors are nearly uncorrelated, since the data overwhelm the prior if sample size increases (in contrast to model selection, as in Rouder & Morey, 2012). Second, Rouder and Morey also standardized the regression coefficients with respect to the scale of the dependent variable. Since the dependent variables are probit-transformed parameters in our case, we only standardize the coefficients with respect to the external covariates. Below, we provide a simulation study to show that these default priors are well calibrated from a frequentist view (e.g., result in unbiased estimates).

In TreeBUGS, covariates are easily included as predictors when fitting a latent-trait MPT model via traitMPT. First, the argument covData that refers to the covariate data needs to be provided, similarly as in the previous section. Second, the argument predStructure determines which regression coefficients are included for which MPT parameters, predStructure = list("a ; pc", "D1 d1 ; age").

Each element of this list starts with one or more MPT parameters and states one or more variables in covData that should be included as predictors after the semicolon. Thereby, predictors are only included for those combinations of MPT parameters and covariates that are of substantive interest. Note that this structure is sufficiently flexible to include predictors that differ within-subjects (e.g., if a covariate changes across two conditions of a source-memory task). For this purpose, repeated measures of the covariate are included as separate columns in covData and can then be assigned to the corresponding MPT parameters (e.g., using the argument predStructure = list("a1;pc1", "a2;pc2", "a3;pc3")).

In our empirical example, we expected the source-guessing parameter to depend on the perceived contingency *pc* in the retrieval condition. In line with this prediction, the credibility interval for the unstandardized regression coefficient did not overlap zero ($\widehat{\beta}=4.56$; 95% CI $=\left[2.74,6.44\right]$). Substantively, this regression coefficient is interpreted as an increase of 0.456 in the latent-probit value of an MPT parameter as perceived contingency *pc* increases by .10.

### Discrete predictors for MPT parameters

In MPT modeling, it is common to test the effect of between-subjects manipulations on the parameters that measure the cognitive processes of interest. Above, we showed that separate latent-trait MPT models can be fitted for each condition in order to compare the group-level means in a second step. However, this procedure results in a rather complex model with separate covariance matrices **Σ**_***1***_, . . . , **Σ**_***I***_ for the *I* conditions. Even though this statistical assumption might be appropriate in some cases, the interest is often only in differences of the group-level means (i.e., differences in the parameter vectors **μ**_***1***_, . . . , **μ**_***I***_), whereas the covariance matrix is assumed to be identical across conditions. Substantively, this means that the hypothesized cognitive states are entered more or less often depending on the condition whereas the parameter correlations across participants remain identical. This nested model with a single covariance matrix **Σ** results in a more parsimonious and specific comparison of mean differences.

To implement this constrained model statistically, we add a linear term on the latent probit scale that shifts the individual parameters depending on the condition. More specifically, we use a design matrix ***X***_***s***_ that indicates the group membership of participants and a vector **η**_***s***_ of effects for the *s*th MPT parameter,

10 $$ {\Phi}^{-1}\left({\uptheta}_{ps}\right)={\upmu}_s+{\boldsymbol{X}}_{\boldsymbol{s}}{\boldsymbol{\upeta}}_{\boldsymbol{s}}+{\updelta}_{ps}. $$

Here, the first summand represents the intercept whereas the second term determines the group-specific deviations from the overall mean. Note that this approach is identical to the standard way of implementing an analysis of variance (ANOVA) within the general linear model (Rouder, Morey, Speckman, & Province, 2012). This model structure results in different means of the MPT parameters across conditions if $\boldsymbol{\upeta}$_***s***_ differs from the null vector, whereas the covariance matrix **Σ** associated with the participant random effects ${\updelta}_{ps}$ remains unaffected.

Without further constraints, the parameter vector $\boldsymbol{\upeta}$_***s***_ is not identifiable. Moreover, sensible priors for $\boldsymbol{\upeta}$_***s***_ are required. With respect to both of these issues, we follow the approach of Rouder et al. (2012), who developed default priors for ANOVA. On the one hand, if the factor has a small number of well-defined levels, a fixed-effects model is assumed by adding the linear constraint that each of the columns of the design matrix **X**_***s***_ sums up to zero (i.e., sum-to-zero coding), which reduces the dimension of the vector $\boldsymbol{\upeta}$_***s***_ by one.^7^ On the other hand, if there are many exchangeable factor levels, a random-effects model is more appropriate, which assumes that the elements of the vector $\boldsymbol{\upeta}$_***s***_ are drawn from independent normal distributions with variance *g*. Similar as for continuous predictors above, the variance parameter g has an inverse *χ* ^2^(1) prior. Note that our priors differ slightly from those of Rouder et al. (2012, p. 363), who standardized the effects $\boldsymbol{\upeta}$_***s***_ with respect to the error variance of the dependent variable.

In TreeBUGS, discrete factors are added using the argument predStructure similarly as for continuous predictors above. If any of the included covariates is recognized as a factor (as indicated by character values), this covariate is automatically added as a discrete fixed-effects predictor. To change this default, the argument predType = c("c","f","r") (using the same order of variables as in covData) allows to define each covariate either as a continuous ("c"), a discrete fixed-effects ("f"), or a discrete random-effects ("r") predictor. Once posterior samples for the model have been obtained by a call to traitMPT, estimates for the group means of the MPT parameters are provided by the function getGroupMeans (including credibility intervals and convergence statistics).

### Data generation and simulations for hierarchical MPT models

The integration of TreeBUGS within R allows the user to easily run Monte Carlo simulations to assess the expected precision of the parameter estimates for a given sample size or to test the influence of different priors. For this purpose, TreeBUGS provides three functions to generate responses for a given set of MPT parameters. Whereas the function genMPT allows to generate response frequencies for any matrix theta of individual MPT parameter values, the functions genBetaMPT and genTraitMPT assume specific hierarchical structures (beta-MPT and latent-trait, respectively) and generate values for the individual MPT parameters based on information about the mean and standard deviations on the group-level. Whereas the latter functions are tailored to standard hierarchical MPT models, the former function allows generating more complex data structures, for instance, for scenarios involving predictors.

As an example of how to run simulations, we provide an R script in the Online Appendix to estimate the precision of the regression coefficients for the memory parameters *d* and *D* of the 2HTSM on the basis of the latent-trait approach. In 500 replications, we generated responses of 50 participants that responded to the same number of items as in our example (i.e., 16 items per source and 32 new items). With the exception of a slightly higher value for recognition memory *D*, we chose data-generating latent probit means (and probit *SD*s) that were similar to the results in the empirical example [i.e., *a* = 0.3 (0.6), *b* = −0.1 (0.5), *d* = 0.6 (1.0), *D* = 0.3 (0.2)]. Data were generated under the assumption that a normally distributed predictor enters the linear probit regression in Eq. 7 with standardized regression coefficients of ${\beta}^D=-0.3$ and ${\beta}^d=0.5$.

Table 2 shows the results of this simulation, based on sampling eight MCMC chains with 10,000 iterations each, of which the first 5,000 samples were discarded, which resulted in good convergence, as indicated by $\widehat{R}<1.05$ for all replications and selective graphical checks. For all parameters, the means of the posterior-mean estimates across simulations were close to the true, data-generating values, resulting in a small absolute bias. Moreover, the data-generating parameters were in the 95% credibility intervals in more than 89% of the replications for all parameters except the mean of *D*, for which this proportion was only 85%. Nevertheless, these results are satisfactory, given that the resulting CIs were relatively small and precise, and considering their nonfrequentist definition as the posterior belief of plausible parameter values (whereas for frequentist *confidence* intervals, these simulated percentages of overlaps should be equal to 95% by definition; Morey, Hoekstra, Rouder, Lee, & Wagenmakers, 2016).

**Table 2:** Parameter recovery simulation of the latent-trait 2HTSM with two predictors

Parameter	Data-Generatin	Means Across Replications				Percentage of Replications	
		Posterior Mean	2.5%	97.5%	Absolute Bias	True in 95% CI	0 Not in 95% CI
Mean *a*	0.62	0.61	0.53	0.69	0.62	95	
Mean *b*	0.46	0.46	0.39	0.53	0.46	95	
Mean *d*	0.73	0.73	0.57	0.88	0.73	95	
Mean *D*	0.62	0.62	0.58	0.65	0.62	85	
*SD* ${\upsigma}_a$	0.60	0.63	0.45	0.85	0.08	94	
*SD* ${\upsigma}_b$	0.50	0.52	0.37	0.71	0.07	94	
*SD* ${\upsigma}_d$	1.00	1.26	0.68	2.23	0.37	89	
*SD* ${\upsigma}_D$	0.20	0.19	0.07	0.31	0.05	95	
Slope ${\beta}^d$	0.50	0.58	0.08	1.25	0.23	95	63
Slope ${\beta}^D$	−0.30	−0.29	−0.39	−0.20	0.05	89	100

Based on 500 replications. Group-level means $\pi \left(\mu \right)$ are on the probability scale whereas the group-level SDs σ and slopes *β* are on the latent probit scale. 95% credibility intervals (i.e., 2.5% and 97.5% posterior quantiles) are computed per replication and then averaged. The absolute bias is computed as the difference between data-generating parameter and posterior mean. The percentage “0 not in 95% CI” is only relevant for the slope parameters to estimate the sensitivity of the hierarchical model to detect a nonzero regression effect.

Of most interest, the slope-parameter estimates were approximately unbiased and sufficiently precise, although ${\beta}^D$ was estimated more precisely than ${\beta}^d$. This was due to the 2HTSM, in which less information is available about the source-memory parameter *d*, because it is defined conditionally on recognition memory *D*. The last column of Table 2 shows that in most replications, the 95% Bayesian credibility intervals did not overlap zero. This indicates the sensitivity of the hierarchical MPT model to detect nonzero regression coefficients using Bayesian *p* values, similar to statistical power in the frequentist framework. Overall, this simulation shows that the proposed default priors on the regression coefficients in the latent-trait MPT model result in desirable frequentist properties of the Bayesian estimates (i.e., unbiasedness and sufficient precision to detect an effect).

Note that our simulation results are only valid for the 2HTSM given a specific set of parameters, and therefore do not generalize to other MPT models, a limitation inherent in any simulation. As a remedy, TreeBUGS provides the user with the necessary methods to run simulations that are tailored to specific MPT models and scenarios of interest.

## General discussion

We provided a nontechnical introduction to the analysis of hierarchical MPT models, which assume that the MPT structure holds for each participant with different parameters. Moreover, we presented the user-friendly R package TreeBUGS that allows researchers to focus on running experiments and analyzing data instead of programming and testing fitting routines. TreeBUGS includes MPT-tailored functions to fit, summarize, and plot parameters and predictions of latent-trait MPT (Klauer, 2010) and beta-MPT models (Smith & Batchelder, 2010). Whereas the former approach explicitly models the covariance of individual MPT parameters by a multivariate normal distribution on the latent probit scale, the latter assumes independent beta distributions. Hence, the latent-trait approach is more appropriate for MPT models including cognitive processes that might be correlated (e.g., item recognition and source memory). Other functions of TreeBUGS include tests for homogeneity of participants or items (Smith & Batchelder, 2008), data generation for simulations, and comparisons of parameter estimates for within- and between-subjects designs.

Moreover, we developed and implemented statistical extensions of the latent-trait MPT approach to include continuous and discrete predictors for the individual MPT parameters. Similar to the generalized linear model (Pinheiro & Bates, 2000), TreeBUGS adds linear terms on the latent probit scale to model the effect of external covariates on the MPT parameters. For continuous predictors, we adapted the weakly informative, scale-invariant priors by Rouder and Morey (2012). In addition to the regression approach, TreeBUGS allows for estimating the population correlation of continuous covariates with the MPT parameters (both in latent-trait and beta-MPT models). This method might be preferable when the parameters are not assumed to be affected by the external covariates, and when many variables are included as with neuro-physiological data (cf. Ly et al., in press).

Regarding discrete predictors, TreeBUGS allows for including between-subjects factors as either fixed or random effects, based on the default priors for ANOVA by Rouder et al. (2012). Note that this approach differs from the standard MPT modeling approach of defining a set of new parameters for each condition (e.g., *D*_*i*_, *d*_*i*_, *a*_*i*_, and *b*_*i*_ for conditions *i* = 1, 2). For hierarchical MPT models, the latter approach requires to fit two or more latent-trait MPT models with separate covariance matrices **Σ**_***i***_. This more complex model structure might not provide sufficient information to estimate to covariance matrix and be prone to overfitting. In contrast, TreeBUGS allows adding a variable that encodes the condition as a fixed or random effect, thereby assuming different group-means of the MPT parameters across conditions but the same covariance structure within each condition. Given that this assumption is valid, the latter approach provides a more precise test of between-subjects manipulations on group-mean parameters.

### Limitations

Currently, TreeBUGS is limited to hierarchical MPT models that either account for participant or item heterogeneity. Given that items can usually be selected to be sufficiently homogeneous (for an example and test, see Smith & Batchelder, 2008), we think that hierarchical models for participant heterogeneity as provided by TreeBUGS are often appropriate. Note that the *χ* ^2^ and permutation test implemented in TreeBUGS can be used to test for item homogeneity (Smith & Batchelder, 2008). If both sources are heterogeneous, it might be necessary to rely on the crossed-random effects approach by Matzke et al. (2015), in which the participant item random effects combine additively on the probit scale (e.g., Rouder & Lu, 2005; Rouder et al., 2007).

Other limitations concern the methodology of hierarchical Bayesian models in general (Lee & Wagenmakers, 2014). First, it is often difficult to judge the relative influence of the prior and the data on the posterior estimate for a given sample size. Ideally, the parameter estimates are mainly informed by the data and not by the prior. Indeed, it is well known that the data overwhelm the prior for large sample sizes (e.g., Rouder et al., 2007). However, it depends on the model structure what a “large” sample size is. Moreover, whereas a large number of participants allows to estimate the group-level parameters precisely, many responses per participant allow to estimate individual parameters precisely. As a remedy for these complex interactions, TreeBUGS facilitates prior-sensitivity simulations for specific models and scenarios.

Second, regarding goodness of fit, the asymptotic distribution of posterior-predictive *p* values (PPP) is neither precisely defined nor known in general (Meng, 1994). Therefore, a criterion indicating a satisfactory goodness of fit such as $p>.05$ needs to be differently interpreted than frequentist *p* values, which underscores the heuristic value of PPP values and contrasts with the precise definition of goodness-of-fit tests in a frequentist framework. As a remedy, model fit can and should also be assessed qualitatively, which is easily done within TreeBUGS by plotting the observed versus the predicted mean frequencies and covariances.

A third limitation concerns the methods to test parameter constraints and select between competing models. To test parameter constraints in between- or within-subjects designs, TreeBUGS only computes credibility intervals of parameter differences and Bayesian *p* values (i.e., the proportion of posterior samples not adhering to the null hypothesis). To select between models, TreeBUGS provides posterior predictive checks of absolute model fit and DIC values. Given that the DIC has been criticized for several shortcomings (e.g., Gelman et al., 2014), it might often be preferable to compute Bayes factors, which quantify the relative evidence in favor of a constraint or in favor of one versus another model (Kass & Raftery, 1995). However, general-purpose methods to compute Bayes factors for hierarchical models are currently not available due to computational limitations.

## Conclusion

The class of MPT models has been very successful in measuring the cognitive processes underlying memory, reasoning, decision making, attitudes, and many other mental characteristics (Erdfelder et al., 2009). To account for individual differences in parameters, we developed the user-friendly software TreeBUGS that facilitates the analysis of hierarchical MPT models. Besides tests of homogeneity, flexible fitting functions, within- and between-subjects comparisons, inclusion of predictors, and MPT-tailored summaries and plots, TreeBUGS first of all enables substantive researchers to think about a new type of hypotheses—that is, theories that explain individual differences when modeling cognitive processes.

## Footnotes

1 The most recent developer version of TreeBUGS is available at https://github.com/denis-arnold/TreeBUGS.

2 The original file format as used by AppleTree or GPT required the number of equations in the first line.

3 TreeBUGS also provides the function simpleMPT for fitting nonhierarchical, Bayesian MPT models to aggregated or individual data (using conjugate beta distributions as priors for the parameters).

4 Technically, this bivariate prior is defined by the probability density function $\pi \left(\alpha, \beta \right)\propto {\left(\alpha +\beta \right)}^{-5/2}$ for *α* and *β* in the interval [0.01, 5,000].

5 Given the parameters μ and σ^2^, TreeBUGS uses numerical integration to compute $E\left[\uppi \left(\mathrm{X}\right)\right]$ and $\mathrm{V}\mathrm{a}\mathrm{r}\left[\uppi \left(\mathrm{X}\right)\right]$ for a random variable $X\sim \mathrm{Normal}\left(\upmu, {\upsigma}^2\right)$.

6 Usually, the scale parameter is referred to by the letter *s*, which is already used for the index of MPT parameters in the present article.

7 By using sum-to-zero coding, TreeBUGS ensures that the priors on the effects are symmetric (for details, see Rouder et al., 2012, p. 363), which is not necessarily the case if simple dummy-coding is used. Currently, this default cannot be adjusted to add custom design matrices.
